# Visual and anatomical failure of anti-VEGF therapy for retinal vascular diseases: a survival analysis of real-world data

**DOI:** 10.1038/s41433-024-03529-9

**Published:** 2024-12-10

**Authors:** Dun Jack Fu, Amit V. Mishra, Chrystie Quek, Konstantinos Balaskas, Nikolas Pontikos, Dawn Sim, Sobha Sivaprasad, Livia Faes

**Affiliations:** 1 NIHR Biomedical Research Centre At Moorfields Eye Hospital NHS Foundation Trust, UCL Institute of Ophthalmology, London, UK; 2 Kings College London, London, UK; 3 Yong Loo Lin School of Medicine, National University of Singapore, Singapore, Singapore; 4 Genentech Roche, South San Francisco, CA USA

**Keywords:** Retinal diseases, Outcomes research

## Abstract

**Importance:**

Predicting undesirable outcomes following anti-VEGF initiation in macular oedema is critical for effective clinical decision-making and optimised care.

**Objective:**

To estimate the time to undesirable events in diabetic macular oedema (DMO), central and branch vein occlusions (CRVO and BRVO) after appropriate loading doses with either ranibizumab or aflibercept and identified baseline predictors of negative outcome.

**Design, setting, participants:**

A retrospective cohort study of 3277 patients (*N* = 2107 in DMO, *N* = 413 in CRVO and *N* = 757 in BRVO) collected over a 10-year period, in a large UK tertiary centre. Only one eye was included per patient. Inclusion criteria pre-specified a minimum of two clinic visits with one being at least 6 months post treatment.

**Main outcome and measures:**

The main outcome measure was absence of visual acuity (VA) improvement due to macular oedema failure of anti-VEGF therapy (defined as VA gain <5 ETDRS letters and CST increase of 50 µm or CST > 325 µm) modelled using time-event analyzes of appropriately loaded patients. Secondary outcomes included survival curves by individual condition (DMO, CRVO, BRVO) and factors associated with negative outcomes.

**Results:**

*After starting anti-VEGF*, there was a 50% chance of undesirable outcomes at 2.3, 5.24 and 6.16 years for DMO, CRVO and BRVO, respectively. Cox proportional hazards modelling identified presenting age, intraretinal (IRF) volume, presence of DMO and VA as predictors of negative outcomes, whilst South East Asian ethnicity conferred an independent protective effect.

**Conclusion:**

Real-world data suggest that undesirable events following anti-VEGF injections is likely to in 50% of patients by the third year of treatment in spite of appropriate loading. The definition of undesirable treatment events captured nearly all patients who were escalated to another therapy, but this proportion represented a small percentage of our definition of failed response.

## Introduction

Macular oedema impairs central vision as a result of fluid accumulation within the macular region of the retina and if left untreated, can lead to permanent vision loss. Macular fluid accumulates in diabetic eye disease (diabetic macular oedema [DMO]) and retinal vein occlusions (RVO) that includes both central retinal vein occlusion (CRVO) and branch retinal vein occlusions (BRVO)—the three common retinal vascular diseases that cause visual impairment [1, 2]. The prevalence of DMO in Europe is 3.7% among people living with diabetes with a projected 30% increase by 2050. Around 16 million people worldwide have been diagnosed with RVO, and an estimated 20% of this population develop oedema [1, 3].

Anti-VEGF therapy is the standard of care for DMO and macular oedema secondary to RVO [4, 5]. Macular oedema secondary to RVO may also be initiated on dexamethasone as a first-line therapy based on clinician and patient choice [6, 7]; yet intravitreal anti-VEGF treatments are safer and superior to all steroids (dexamethasone implant, triamcinolone injection) and non-steroid therapies (laser photocoagulation) in optimising outcomes of visual acuity within macular oedema secondary to retinal vascular diseases [8–10]. The annual demand on anti-VEGF therapy services is high and increasing: Moorfields Eye Hospital administered 8620 injections in 2019 for DMO and 7143 for RVO, and this is projected to increase to 15,900 and 13,176 by 2029, respectively [11]. Most of these injection schedules have a significant burden to the patients in terms of anxiety and quality of life [12].

In a multicentre randomised clinical trial comparing intravitreal aflibercept, bevacizumab and ranibizumab for vision-impairing centre-involved DMO (DRCR.net protocol T), 31.6% of patients on aflibercept and 41.5% on ranibizumab experienced persistent oedema following 24 weeks of treatment, indicating that non-response to anti-VEGF monotherapy is not uncommon [13]. More than 44% of these patients maintained increased central retinal thickness (CRT) at the end of the second year of therapy [14–17]. The prevalence and individual likelihood of anti-VEGF failure in DMO or RVO is currently unknown. This is in part due to a range of terms (‘persistent’, ‘refractory’, ‘chronic’) that are used to describe lack of improvement in CRT or best corrected visual acuity (BCVA). Definitions of poor response may rely on various CRT thresholds, or a combination of BCVA and CRT measures. The absence of consensus definitions hinders study and comprehension of anti-VEGF treatment failure; which in turn adversely affects investigation of switch to alternative treatments, as well as, appropriate implementation of alternative therapies in clinical practice.

Here we examined 10 years of data collected as part of routine clinical care to better understand undesirable outcomes following initiation of anti-VEGF therapy. We used Kaplan–Maier survival analysis as well as Cox regression modelling to identify predictive factors for missing defined treatment goals. In an effort to define and explore the diminishing benefits of anti-VEGF therapy, we have considered multiple key definitions from the existing literature, employing a composite definition that includes both functional and anatomical parameters to capture vision loss due to persistence of oedema, and by inference absence of response to anti-VEGF.

## Methods

### Study design and setting

This retrospective cohort study utilized electronic medical records of patients attending a tertiary London hospital, Moorfields Eye Hospital NHS Foundation Trust, between 1 January 2012 and 1 April 2022. The study was conducted in compliance with the Declaration of Helsinki. This investigator-initiated study was approved by the United Kingdom Research Ethics Committee (REC 21/NE/0193) and the service evaluation was registered in Moorfields Eye Hospital Clinical Audit Department (CA21/MR/1026). It is written in line with the Strengthening the Reporting of Observational Studies in Epidemiology reporting guideline. Informed consent from the study cohort was not required as per the standard when using retrospective, de-identified data for research within NHS England.

### Cohort

The cohort comprised treatment-naïve patients with DMO and macular oedema secondary to CRVO or BRVO that were initiated on anti-VEGF therapy (Supplementary Fig. 1). Patients were excluded if they were younger than 18 years; had incomplete loading phase (appropriate loading defined as receiving three initial injections with an inter-injection duration of 1 month ± 10 days); had insufficient follow-up (fewer than two ophthalmic visits following initial injection with at least one falling beyond 6 months); missing VA measurements or OCT imaging at baseline; previous intraocular or periocular steroid treatment. Patients were started on the Moorfields Eye Hospital anti-VEGF therapy regimen approved by the Clinical Audit and Effectiveness Committee and described previously, namely an initial loading phase followed by treat and extend [18]. Socioeconomic status of the patient was measured using the English index of multiple deprivation [19].

### Automatic segmentation of macular oedema features from OCT

**Fig. 1:**
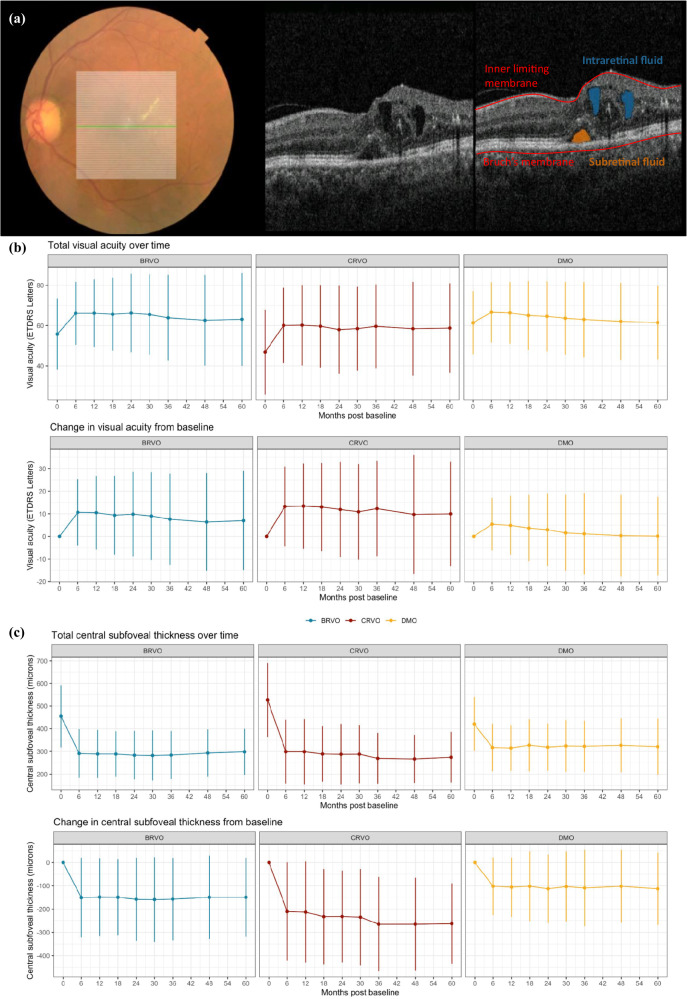
Macular oedema features. **a** Automatic segmentation of macular oedema features. Exemplar model output on a full OCT volume for a given eye macular oedema. colour fundus photo (left panel) with en-face projection of OCT B-scans. A single B-scan (indicated with a green line) shown without (middle panel) and with (right panel) segmentation overlay for intraretinal fluid (blue) and subretinal fluid (orange). Volume in nanolitres is the sum of all voxels with a given feature present multiplied by voxel volume. Fovea and retinal sublayers inner limiting membrane and Bruch’s membrane were segmented using the Topcon Advanced Boundary Segmentation Tool (TABS) version 1.6.2.6, software. Central subfoveal thickness in microns was calculated as mean total retinal thickness (inner limiting membrane to Bruch’s membrane) within a 1 mm diameter circle centred on the fovea. Mean absolute values and change from baseline of **b** visual acuity measured in Early Treatment Diabetic Retinopathy Study (ETDRS) letters and **c** central subfoveal thickness (CST; µm) following initiation of anti-VEGF in patients with diabetic macular oedema (DMO; yellow), central retinal vein occlusion (CRVO; red) and branch retinal vein occlusion (BRVO; red) up to 5 years. Each timepoint allowed a margin of 60 days. Error bars signify standard deviation.

Eyes were scanned with either a Topcon 3D OCT-1000 or a Topcon 3D OCT-2000 (Topcon Corporation, Tokyo, Japan), resulting in OCT volumes with a resolution of either 512 × 128 A-scans or 256 × 256 A-scans, covering a macular area of 6 mm × 6 mm. Intraretinal and subretinal fluid volume (nL) was quantified using a previously published automatic segmentation model (Fig. 1a) [20]. Central subfoveal thickness (CST) was calculated as the mean total retinal thickness (distance between inner limiting membrane and Bruch’s membrane) within a 1 mm diameter circle centred on the fovea.

### Study outcomes

Moorfields Eye Hospital adheres to the National Institute for Health and Care Excellence (NICE) guidance (TA346, TA274, TA283, TA305, TA283), which recommends anti-VEGF therapy only if visual impairment caused by macular oedema is present. The aim of treatment initiation is therefore to improve or stabilise visual outcomes, and it therefore follows that an undesirable event following initiation of anti-VEGF therapy is the absence of visual improvement or loss of vision. Accordingly, the primary outcome was the median event duration between initiating anti-VEGF therapy and an undesirable treatment event defined as either: absence of VA improvement due to macular oedema (CST > 325 µm and VA change from baseline of <5 ETDRS letters) at 2 consecutive visits or loss of VA attributable to increase in macular oedema (CST increase by 50 µm and loss of at least 10 ETDRS letters from baseline). These are composite definitions drawing on the PHOTON trial and TREX-DMO trial definitions of persistent DMO which jointly considers visual and anatomical outcomes [21, 22]. This was further sub-stratified according to pathology (DMO, BRVO, CRVO). Multivariable Cox regression was carried out to extrapolate hazard ratios (HR) for factors associated with anti-VEGF failure. Cox proportional hazards models were used to relate visual outcomes to both time-independent (age at baseline, VA at baseline, sex, ethnicity, intraretinal fluid at baseline, subretinal fluid at baseline, indication) and time-dependent (anti-VEGF injections given up until the point of event, thereby censoring any injection beyond event timepoint) clinical covariates. An available case analysis was carried out to report mean VA and CST at 6 monthly timepoints following baseline.

### Statistical analysis

Hazards were modelled with Kaplan–Meier models [23]. Survival curves were plotted using the classical Kaplan–Meier estimator based on tabulation of the number at risk and number of events at each unique event time. In comparison to RCTs, real-world cohorts feature a considerable number of absent values at monthly time points as monthly examinations are not protocolized as in RCTs (Supplementary Fig. 2). However, the data for a given patient are only missed with such an available case analysis. For instance, 20% (635/3227) of eyes did not have a VA measurement at the 12-month timepoint (365 ± 60 days) yet 100% had a VA measurement following the 12-month timepoint. Time-event analyzes were thus selected to inform the primary outcome as they make use of all data that is available and obviate the survival bias encountered in traditional visual outcome at given time point metrics (e.g. mean change in VA at year 1). Moreover, by incorporating all the available data leading up to missing values in our models for VA outcomes, they ought to reflect real life more accurately.

In the UK, NICE recommends intravitreal steroids for DMO and macular oedema secondary to BRVO and CRVO if the condition has not responded well enough to anti-VEGF therapy (TA349 and TA229). Thus, to account for potential effects of steroids, data were censored at the point of escalation to steroid therapy and cataract surgery. All clinical data were recorded within an electronic medical record application (OpenEyes Foundation), as previously described [24]. All data analyzes were carried out with R (version 3.5.1) [25].

## Results

### Cohort and baseline characteristics

Between 1 January 2012 and 1 April 2022, 3277 eyes of 2742 patients met the study criteria. The cohort comprised 3277 eyes with macular oedema initiated on anti-VEGF therapy secondary to DMO (2107), CRVO (413), or BRVO (757) (Supplementary Fig. 1). Baseline demographic and clinical characteristics are summarised in Tables 1 and 2, respectively.

**Table 1:** Baseline demography of study cohorts stratified by treatment indication.

Baseline patient-level demographics				
	DMO (*N* = 1600)	CRVO (*N* = 401)	BRVO (*N* = 741)	Overall (*N* = 2742)
Gender				
Male	965 (60.3%)	224 (55.9%)	368 (49.7%)	1557 (56.8%)
Female	634 (39.6%)	177 (44.1%)	373 (50.3%)	1184 (43.2%)
Age at recruitment				
Mean (SD)	63 (11)	68 (14)	69 (12)	65 (12)
Median (IQR)	63 (14)	70 (19)	69 (17)	66 (16)
Ethnicity				
Afrocarribean	173 (10.8%)	29 (7.2%)	73 (9.9%)	275 (10.0%)
Caucasian	336 (21.0%)	175 (43.6%)	263 (35.5%)	774 (28.2%)
Chinese	4 (0.3%)	4 (1.0%)	4 (0.5%)	12 (0.4%)
Mixed	20 (1.3%)	4 (1.0%)	3 (0.4%)	27 (1.0%)
South-East Asian	537 (33.6%)	50 (12.5%)	136 (18.4%)	723 (26.4%)
Unknown	529 (33.1%)	139 (34.7%)	262 (35.4%)	930 (33.9%)
Index of multiple deprivation decile				
1	52 (3.3%)	18 (4.5%)	14 (1.9%)	84 (3.1%)
2	273 (17.1%)	48 (12.0%)	77 (10.4%)	398 (14.5)
3	310 (19.4%)	52 (13.0%)	93 (12.6%)	455 (16.6%)
4	251 (15.7%)	46 (11.5%)	95 (12.8%)	392 (14.3%)
5	182 (11.4%)	46 (11.5%)	91 (12.3%)	319 (11.6%)
6	166 (10.4%)	43 (10.7%)	98 (13.2%)	307 (11.2%)
7	127 (7.9%)	38 (9.5%)	70 (9.4%)	235 (8.6%)
8	108 (6.8%)	42 (10.5%)	65 (8.8%)	215 (7.8%)
9	74 (4.6%)	39 (9.7%)	82 (11.1%)	195 (7.1%)
10	51 (3.2%)	28 (7.0%)	50 (6.7%)	129 (4.7%)
Null	5 (0.3%)	1 (0.2%)	6 (0.8%)	12 (0.4%)
Missing	1 (0.1%)	0 (0.0%)	0 (0.0%)	1 (0.0%)

Mean, median, standard deviation (SD) and interquartile range (IQR) are shown for demographic characteristics at baseline. Baseline time point was taken to be time at initiation of intravitreal anti-VEGF therapy.

*DMO* diabetic macular oedema, *CRVO* central retinal vein occlusion, *BRVO* branch retinal vein occlusion.

**Table 2:** Clinical features at baseline.

Baseline eye-level clinical features				
	DMO (*N* = 2107)	CRVO (*N* = 413)	BRVO (*N* = 757)	Overall (*N* = 3227)
Baseline VA (ETDRS letters)				
Mean (SD)	61 (16)	47 (21)	56 (17)	58 (17)
Median (IQR)	65 (18)	50 (27)	59 (24)	61 (20)
Baseline VA greater than or equal to 70 ETDRS letters				
<70	1367 (64.9%)	357 (86.4%)	569 (75.2%)	2293 (70.0%)
70 or over	740 (35.1%)	56 (13.6%)	188 (24.8%)	984 (30.0%)
Baseline central subfoveal thickness (microns)				
Mean (SD)	420 (120)	510 (170)	440 (140)	430 (140)
Median (IQR)	400 (140)	510 (240)	430 (200)	410 (170)
Intraretinal fluid (nL)				
Mean (SD)	76 (85)	150 (150)	130 (120)	97 (110)
Median (IQR)	47 (86)	110 (180)	91 (140)	62 (110)
Subretinal fluid (nL)				
Mean (SD)	80 (350)	380 (730)	340 (660)	180 (510)
Median (IQR)	0.12 (15)	47 (430)	25 (390)	1.4 (67)
Phakic status				
Aphakic	3 (0.1%)	0 (0.0%)	1 (0.1%)	4 (0.1%)
Phakic	1170 (55.5%)	315 (76.3%)	562 (74.2%)	2047 (62.5%)
Pseudophakic	564 (26.8%)	86 (20.8%)	174 (23.0%)	824 (25.2%)
Missing	370 (17.6%)	12 (2.9%)	20 (2.6%)	402 (12.3%)
Anti-VEGF agent				
Aflibercept	1355 (64.3%)	385 (93.2%)	514 (67.9%)	2254 (68.8%)
Ranibizumab	752 (35.7%)	28 (6.8%)	243 (32.1%)	1023 (31.2%)

Baseline time point was taken to be time at initiation of intravitreal anti-VEGF therapy.

*DMO* diabetic macular oedema, *CRVO* central retinal vein occlusion, *BRVO* branch retinal vein occlusion, *VA* visual acuity, *ETDRS* early treatment diabetic retinopathy study.

### Change in vision and subfoveal thickness following initiation of anti-VEGF therapy

Following initiation of anti-VEGF therapy, mean VA increased in all three groups (Fig. 1b). At 6 months the mean VA change from baseline was 13 (standard deviation [SD] 17) ETDRS letters in the CRVO cohort, followed by the BRVO (10 [16] ETDRS letters) and DMO cohorts (5 [SD 11] ETDRS letters) (Fig. 1b, Supplementary Table 1). Mean CST decreased following initiation of anti-VEGF treatment, in a similar pattern to VA, with CRVO group showing the greatest nominal mean decrease in CST (Fig. 1b). At Month 6, mean CST in CRVO group was 300 (SD 140) µm, a mean change from baseline of −210 (SD 210) µm (Supplementary Table 1). Notably, the standard deviations around group means at each time point are considerable. This indicates heterogeneity of treatment response amongst patients. For example, the mean VA gain at month 12 for patients with DMO (4.9 [SD 13] ETDRS letters) suggests an overall improvement across the sub-cohort; but also demonstrates that a proportion of patients lose VA. Therefore, we next directly queried these undesirable clinical outcomes of anti-VEGF therapy.

### Likelihood and time to undesirable outcomes following anti-VEGF therapy

**Fig. 2:**
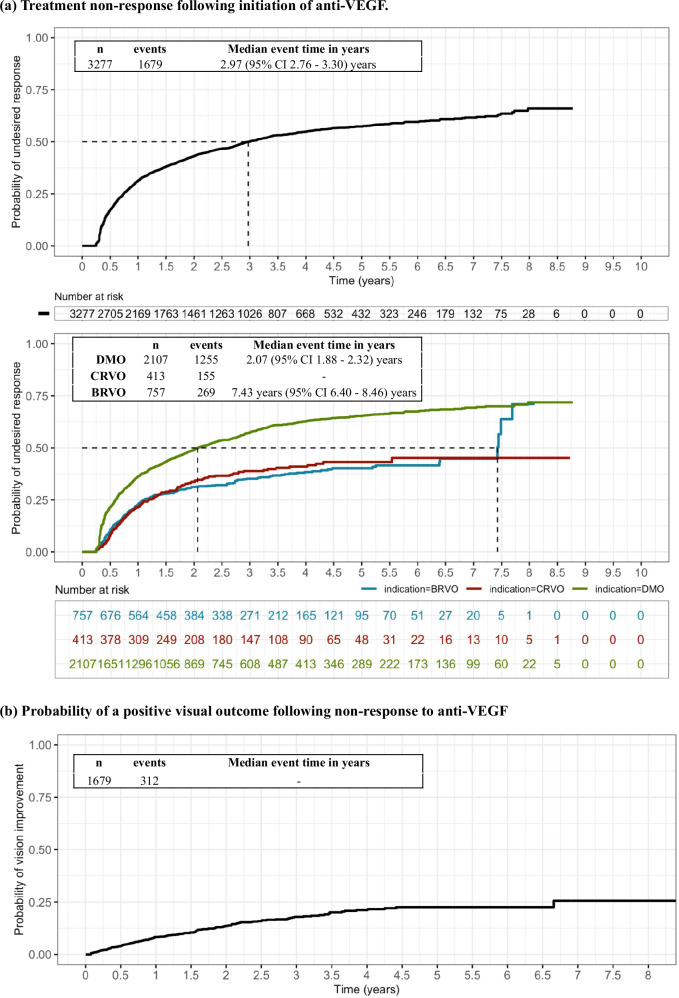
Time to treatment outcomes. **a** Probability of an undesired treatment outcome (absence of VA improvement or VA-loss attributable to macular oedema) following initiation of anti-VEGF. Kaplan–Meier modelling was carried out to estimate time to treatment failure (one of either: visual acuity (VA) gain less than 5 early treatment diabetic retinopathy study (ETDRS) letters with central subfoveal thickness (CST) 325 µm or more at 2 consecutive visits; VA loss of 10 ETDRS letters and CST increase of 50 µm; or escalation to steroid therapy) sub-stratified by each of the treatment indications (DMO, green; CRVO, red; BRVO, blue; bottom panel). Remaining numbers at risk shown below graph. **b** Probability of a positive visual outcome following an undesired treatment outcome. Kaplan–Meier modelling was carried out to estimate time to visual acuity gain of 5 early treatment diabetic retinopathy study (ETDRS) letters or more from baseline (initiation of anti-VEGF) following an undesirable treatment outcome sub-stratified by each of the treatment indications (DMO, green; CRVO, red; BRVO, blue; bottom panel). Data were censored following escalation to intravitreal steroid or cataract surgery.

Here, undesirable events following anti-VEGF treatment were defined as absence of VA improvement due to macular oedema (CST > 325 µm and VA change from baseline <5 ETDRS letters) at 2 consecutive visits or loss of VA attributable to increase in macular oedema (CST increase by 50 µm and loss of at least 10 ETDRS letters from baseline). This is a composite definition drawing on the PHOTON trial and TREX-DMO trial definitions of persistent DMO which jointly considers visual and anatomical outcomes. Overall, 1679 of the 3277 study eyes (51%) experienced absence of VA improvement or VA-loss attributable to macular oedema following anti-VEGF initiation. Kaplan–Meier analysis was carried out to estimate the likelihood of this undesirable outcome. After initiating anti-VEGF treatment, the median time to absence of VA improvement or VA-loss attributable to macular oedema was 2.30 (95% CI 2.03–2.58) years in the DMO group, 5.24 (95% CI 3.73–6.75) years in the CRVO group, and 6.16 (95% CI 5.45–6.87) years in the BRVO group (Fig. 2a).

These results can also be expressed as a probability value. In other words, for those that remain on anti-VEGF therapy, there is a 25% chance of this undesirable outcome at 9 months following initiation of treatment; over 50% at 3 years and nearly 70% at 8 years. In the disease-group sub-analyzes, the probability reached 50% by 2.07 years in those with DMO, but this point is not reached until 5 and 6 years for BRVO and CRVO cohorts, respectively (Fig. 2a).

We next considered whether the definition for an undesirable treatment event used here (absence of VA improvement or VA-loss attributable to macular oedema) could be a transient fluctuation in the course of treatment or be indicative of a more chronic state. Thus, the likelihood of gaining 5 ETDRS letters from anti-VEGF initiation or more after meeting our undesirable outcome criteria was queried. The majority of patients (85% [947/1116] with DMO; 61% [88/144] with CRVO; 75% [174/233] with BRVO) were unable to gain 5 or more ETDRS letters from baseline VA up to 8 years of the remaining observation period (Fig. 2b). Moreover, inadequate response to anti-VEGF is a prerequisite for escalation to intravitreal dexamethasone implant (NICE TA349 and TA229). Of the entire cohort (*n* = 3277), 238 patients were escalated to second-line dexamethasone treatment within the observation period, and 237 (99.6%) were included in the 1679 patients who experienced our definition of undesirable treatment outcome. Of the 312 patients that did experience a positive visual outcome, only 1 patient underwent cataract surgery in the preceding 3 months and thus ascribable to benefit of the surgery.

Sub-analyzes were carried out to consider the individual contributions of the composite outcome and suggest this undesirable treatment event was largely driven by a lack of VA improvement due to macular oedema (CST > 325 µm and VA change from baseline <5 ETDRS letters at 2 consecutive visits) rather than loss of VA attributable to increase in macular oedema (CST increase by 50 µm and loss of at least 10 ETDRS letters from baseline) (Supplementary Fig. 3).

### Clinical biomarkers as predictors of undesirable treatment outcome

Cox proportional hazards models were used to identify covariables predictive of eyes with absence of VA improvement or VA-loss attributable to macular oedema following anti-VEGF therapy (Table 3). Statistically significant associations were detected between likelihood of this undesirable treatment event and baseline age (hazard ratio [HR], 1.19 per 10 years of age; 95% CI 1.13–1.24; *P* < 0.001), baseline VA (HR 1.10 per 5 ETDRS letter score; 95% CI 11–1.12; *P* < 0.001), baseline IRF (HR 1.05 per 50 nl; 95% CI 1.03–1.08; *P* < 0.001), number of anti-VEGF injections (HR 1.05 per injection; 95% CI 1.03–1.06; *P* < 0.001) and disease indication. In comparison to initiating anti-VEGF due to DMO, there was 53% (HR 0.46; 95% CI 0.39–0.53; *P* < 0.001) and 47% (HR 0.51; 95% CI 0.43–0.62; *P* < 0.001) less chance of experiencing an unideal clinical outcome with macular oedema secondary to BRVO and CRVO, respectively (Table 3 and Supplementary Table 2).

**Table 3:** Multivariable associations with anti-VEGF therapy treatment failure.

	Hazard ratio	95% LCI	95% UCI	*P*-value
Sex				
Female	0.93	0.85	1.03	0.18
Age	1.19	1.13	1.24	<0.001
Ethnicity				
Caucasian	1.02	0.86	1.22	0.80
Chinese	1.40	0.65	2.98	0.39
Mixed	0.87	0.54	1.39	0.56
Southeast Asian	0.83	0.79	0.99	0.04
Unknown	0.92	0.78	1.09	0.34
Visual acuity at baseline	1.10	1.08	1.12	<0.001
Intraretinal fluid at baseline	1.05	1.03	1.08	<0.001
Subretinal fluid at baseline	1.00	0.99	1.00	0.68
Anti-VEGF injection	1.05	1.03	1.06	<0.001
Indication				
BRVO	0.46	0.39	0.53	<0.001
CRVO	0.51	0.43	0.62	<0.001

Cox proportional hazards regressions modelling was carried out to query predictive covariates of treatment failure (one of either: visual acuity (VA) gain less than 5 early treatment diabetic retinopathy study (ETDRS) letters with central subfoveal thickness (CST) 325 µm or more at 2 consecutive visits; VA loss of 10 ETDRS letters and CST increase of 50 µm; or switch to steroid therapy) after starting anti-VEGF for DMO, CRVO or BRVO. Hazard ratios alongside 95% confidence intervals presented for: sex; baseline age (per 10 years); ethnicity; baseline visual acuity (per 5 early treatment diabetic retinopathy study letters); baseline intraretinal fluid (per 50 nl); baseline subretinal fluid (50 nl); treatment indication (BRVO or CRVO in comparison to DMO); and anti-VEGF injections (included as a cumulative, time-varying covariate).

## Discussion

Here, evidence is provided that treatment for macular oedema with anti-VEGF injections may fail to provide sufficient clinical benefit for over 25% of patients by 9 months of treatment, 50% by 3 years and nearly 70% by 8 years in spite of appropriate loading. This was strongly driven by the DMO study cohort, with the RVO cohorts experiencing benefits for longer. Patients with DMO were more than twice as likely to experience the absence of VA improvement or VA-loss attributable to macular oedema compared to either CRVO or BRVO.

A consensus definition of treatment non-response for macular oedema does not exist currently. Limited responses to treatment for macular oedema of any cause may be defined using functional and anatomical parameters. Limited functional responses have been defined as VA gains of ≤5 ETDRS letters [26, 27] or <5 ETDRS letters [28–30]. Limited anatomical responses were previously defined as reduction in CST ≤ 20% [26, 27] or as <10% decrease or increase in CST from baseline [29]. Of note, post-hoc analyzes of clinical trials that evaluate limited responses to anti-VEGF therapy usually focus on the functional responses i.e. VA outcomes [28, 30]. Considering VA outcomes in isolation could be confounded by factors unrelated to anti-VEGF itself e.g., vitreous haemorrhage. The findings presented here provide support for the use of a composite definition that includes an anatomical marker, as well as scenarios of limited VA gain or VA loss. If we consider a switch to second-line dexamethasone as a clinical marker of confirmed treatment failure, our definition was sensitive enough to capture all but one patient who was diagnosed as a refractory case by their treating ophthalmologist and therefore eligible for a therapy change. And critically, we report that for patients with DMO or macular oedema secondary to RVO, a good visual outcome is very unlikely from a point of an occurrence of non-response. Remaining on anti-VEGF after this event confers less than 25% chance of ever gaining 5 or more ETDRS letters. This illustrates the benefit of defining treatment failure by quantifying the probability of treatment success if current treatment is unchanged; rather than criteria of arbitrary thresholds.

The aims of defining a limited response are to specify follow-up and retreatment regimens for anti-VEGF treatment [31, 32] and identify patients with initial refractory disease so that alternative therapeutics that may work can be considered [26, 27, 29]. Follow-up periods rarely extend to more than two years. Studies with long-term outcome data are limited. The DRCR Network Protocol T Extension Study including 317 patients with DMO reported treatment and clinical outcomes at 5 years after initiation of anti-VEGF therapy [33]. They found that mean VA worsened between 2 and 5 years in the study cohort. While at the end of year 2, 84% of patients had Snellen 20/40 vision or better (increased from 51% at baseline), only 73% had Snellen 20/40 vision or better at year 5. While a positive mean change from baseline was still reported at year 5 across the treatment groups, VA had decreased by a mean of 5 ETDRS letters across the study population compared to year 2. VA gain from baseline of <5 ETDRS letters was reported in ≥35% of patients for each treatment group, meeting one of our treatment outcome criteria. The study did not report how many patients in this subgroup were still receiving anti-VEGF treatment. Interestingly, mean CRT was similar at 2 and 5 years. Overall, our findings are consistent with data reported in the DRCR Network Protocol T Extension Study, but our results suggest that there may be a higher proportion of patients experiencing treatment failure in the real world than what is suggested in this RCT extension study. Indeed, a Belgian study reporting real-world outcomes in 55 patients with DMO treated with ranibizumab ≥3 years (VISION study) also reported a strong variance in VA response across their study population over time [34]; and a Swiss study with 117 DMO patients initiating treatment with ranibizumab or aflibercept found a downward trend for VA over 5 years, similar to our report [35]. In agreement with results from the DRCR Network Protocol T Extension Study, CRT in this cohort also responded well to treatment and was stable between three and ten years of follow-up. Crucially, none of these studies included an analysis of overall median survival time to a limited response or treatment switch.

We identified age, VA, IRF and therapeutic indication as predictive baseline factors of the absence of VA improvement or VA-loss attributable to macular oedema following initiation of anti-VEGF. Age and baseline VA have previously been suggested as factors in predicting treatment response [36, 37]. With manual OCT segmentation, IRF is commonly reported through binary categorisation (presence or absence) within a scan. The approach used here utilized a machine learning model to automatically quantify OCT markers, thus considering them as continuous variables. Here we observe that for every 50 nl of IRF at treatment initiation, there is an additional 6% (95% CI 1.0–519.0%) greater chance of an undesirable treatment outcome. In comparison to macular oedema related to BRVO and CRVO, DMO was almost twice as likely to experience the absence of VA improvement or VA-loss attributable to macular oedema. Interestingly, at treatment initiation, the DMO sub-cohort was observed to have both greater VA and lower CRT than BRVO and CRVO sub-cohorts. Whilst multiple pathological mechanisms are at play in each of the three disease entities, a possible explanation for this is that the VEGF-pathway plays a greater role in macular oedema secondary to BRVO and CRVO than in DMO—albeit this hypothesis requires support from direct interrogation.

This study features key limitations that ought to be recognised. The definition for ‘absence of VA improvement due to macular oedema’ utilized here does not consider potential determinants of VA that are independent of macular oedema. For instance, macular ischaemia can account for absence of VA improvement or VA loss, regardless of whether macular oedema is present. To account for this, future studies could incorporate additional investigations to adjust for macular ischaemia, for example using OCT angiography. Whilst Kaplan–Meier time-event analyzes were used in this study, smoothing statistics or generalised additive modelling would also be able to represent VA outcomes while taking all available data into consideration and should also be considered in future analyzes.

The interval for VA measurement is typically in monthly units, yet patients can report subjective improvement of vision following anti-VEGF injection that declines over time. If this were to occur over less than a month, then monthly VA measurements would represent the VA trough and potentially miss the extent to which anti-VEGF therapy can improve VA. As such, studies aimed at reporting positive treatment outcomes could consider more frequent VA measurements.

This retrospective cohort study of 3277 patients receiving anti-VEGF treatment for macular oedema suggests that undesirable treatment outcomes (absence of VA improvement or VA-loss attributable to macular oedema) following anti-VEGF initiation is common in the real world and can be expected to occur in about half the patient population by the third year of treatment, in spite of appropriate loading. As anti-VEGF injections are not without risk and are associated with a measurable burden to patients and services, defining anti-VEGF treatment non-response in macular oedema is critical for optimised care and effective clinical decision-making.

## Summary

### What was known before

- What is the likelihood of treatment non-response to anti-VEGF in macular oedema secondary to diabetic macular oedema (DMO), branch retinal vein occlusion (BRVO) and central retinal vein occlusion (CRVO)?

### What this study adds

- From analysis of a retrospective cohort of 3277 patients, non-response was common, occurring in about half the patient population by the third year of treatment, in spite of appropriate loading. We propose and validate an approach to defining treatment non-response. Anti-VEGF injections are not without risk and are associated with a measurable burden to patients and services, thus defining treatment non-response is critical for optimal individualized patient care.

## Supplementary information

Supplementary Table 1. Six-monthly change in visual acuity and central subfoveal thickness from baseline up to five years following anti-VEGF initiation.

Supplementary Table 2. Baseline features stratified by anti-VEGF therapy treatment failure.

Supplementary Fig. 1. CONSORT flow diagram

Supplementary Fig. 2. Data capture at monthly timepoints

Supplementary Fig. 3. Individual contributions of the treatment non-response criteria used in this model.

Supplementary Figs. and Tables Caption

## Data Availability

Data are available as per the terms of Data Sharing Agreement within the Data Access Request Service outlined by NHS Digital.
