# Supplementary material for: Visual and anatomical failure of anti-VEGF therapy for retinal vascular diseases: a survival analysis of real-world data
Source: Eye (Lond). 2024 Dec 10;39(5):977–85. doi: 10.1038/s41433-024-03529-9 (PMC11933433; doi:10.1038/s41433-024-03529-9)
Supplement: Supplementary file 1 — Supplementary Table 1. Six-monthly change in visual acuity and central subfoveal thickness from baseline up to five years following anti-VEGF initiation. [file 41433_2024_3529_MOESM1_ESM.docx]

|  | BRVO | | | | | | | | | |
| --- | --- | --- | --- | --- | --- | --- | --- | --- | --- | --- |
|  | Baseline (N=746) | Month 6 (N=686) | Month 12 (N=685) | Month 18 (N=587) | Month 24 (N=481) | Month 30 (N=436) | Month 36 (N=356) | Month 48 (N=255) | Month 60 (N=158) |  |
| **Visual acuity in ETDRS letters** |  |  |  |  |  |  |  |  |  |  |
| Mean (SD) | 55.8 (17.4) | 66.1 (15.5) | 66.1 (16.7) | 65.6 (17.9) | 66.1 (19.3) | 65.5 (19.7) | 63.8 (21.1) | 62.6 (22.3) | 63.0 (22.9) |  |
| Median [Min, Max] | 59.0 [0, 90.0] | 69.0 [2.00, 95.0] | 70.0 [0, 100] | 70.0 [0, 94.0] | 70.0 [0, 100] | 70.0 [0, 95.0] | 70.0 [0, 94.0] | 70.0 [0, 95.0] | 70.0 [0, 90.0] |  |
| **Change in visual acuity from baseline** |  |  |  |  |  |  |  |  |  |  |
| Mean (SD) | 0 (0) | 10.6 (14.5) | 10.5 (16.1) | 9.35 (17.3) | 9.83 (18.5) | 8.99 (19.2) | 7.61 (20.0) | 6.39 (21.5) | 7.00 (21.8) |  |
| Median [Min, Max] | 0 [0, 0] | 10.0 [-53.0, 73.0] | 10.0 [-81.0, 80.0] | 10.0 [-81.0, 71.0] | 10.0 [-73.0, 80.0] | 9.00 [-81.0, 75.0] | 9.00 [-65.0, 76.0] | 8.00 [-71.0, 62.0] | 9.50 [-71.0, 62.0] |  |

| **(a) Visual acuity in ETDRS letters** | | | | | | | | | | |
| --- | --- | --- | --- | --- | --- | --- | --- | --- | --- | --- |
|  | DMO | | | | | | | | | |
|  | Baseline (N=2107) | Month 6 (N=1914) | Month 12 (N=1844) | Month 18 (N=1606) | Month 24 (N=1444) | Month 30 (N=1352) | Month 36 (N=1205) | Month 48 (N=1001) | Month 60 (N=720) |  |
| **Visual acuity in ETDRS letters** |  |  |  |  |  |  |  |  |  |  |
| Mean (SD) | 61 (16) | 67 (15) | 66 (15) | 65 (17) | 65 (17) | 64 (18) | 63 (18) | 62 (19) | 61 (18) |  |
| Median (IQR) | 65 (18) | 70 (16) | 70 (16) | 70 (21) | 70 (21) | 70 (21) | 70 (21) | 68 (21) | 65 (20) |  |
| **Change in visual acuity from baseline** |  |  |  |  |  |  |  |  |  |  |
| Mean (SD) | 0 (0) | 5.4 (11) | 4.9 (13) | 3.7 (15) | 3.0 (16) | 1.6 (17) | 1.1 (18) | 0.33 (18) | 0.14 (17) |  |
| Median (IQR) | 0 (0) | 5.0 (11) | 5.0 (13) | 4.0 (15) | 4.0 (15) | 3.0 (15) | 2.0 (17) | 2.0 (17) | 2.0 (18) |  |
|  | CRVO | | | | | | | | | |
|  | Baseline (N=413) | Month 6 (N=389) | Month 12 (N=379) | Month 18 (N=327) | Month 24 (N=281) | Month 30 (N=251) | Month 36 (N=195) | Month 48 (N=145) | Month 60 (N=86) |  |
| **Visual acuity in ETDRS letters** |  |  |  |  |  |  |  |  |  |  |
| Mean (SD) | 47 (21) | 60 (18) | 60 (20) | 60 (20) | 58 (22) | 58 (21) | 60 (21) | 58 (23) | 59 (22) |  |
| Median (IQR) | 50 (28) | 63 (26) | 64 (28) | 63 (27) | 62 (28) | 62 (23) | 64 (22) | 62 (29) | 64 (23) |  |
| **Change in visual acuity from baseline** |  |  |  |  |  |  |  |  |  |  |
| Mean (SD) | 0 (0) | 13 (17) | 13 (19) | 13 (19) | 12 (21) | 11 (21) | 12 (21) | 9.7 (26) | 10 (23) |  |
| Median (IQR) | 0 (0) | 11 (19) | 12 (21) | 11 (23) | 11 (23) | 11 (22) | 12 (23) | 11 (29) | 12 (29) |  |
|  | BRVO | | | | | | | | | |
|  | Baseline (N=757) | Month 6 (N=686) | Month 12 (N=685) | Month 18 (N=587) | Month 24 (N=481) | Month 30 (N=436) | Month 36 (N=356) | Month 48 (N=255) | Month 60 (N=158) |  |
| **Visual acuity in ETDRS letters** |  |  |  |  |  |  |  |  |  |  |
| Mean (SD) | 56 (17) | 66 (15) | 66 (17) | 66 (18) | 66 (19) | 65 (20) | 64 (21) | 63 (22) | 63 (23) |  |
| Median (IQR) | 59 (25) | 69 (17) | 70 (21) | 70 (24) | 70 (21) | 70 (20) | 70 (23) | 70 (28) | 70 (24) |  |
| **Change in visual acuity from baseline** |  |  |  |  |  |  |  |  |  |  |
| Mean (SD) | 0 (0) | 11 (15) | 10 (16) | 9.3 (17) | 9.8 (19) | 9.0 (19) | 7.6 (20) | 6.4 (21) | 7.0 (22) |  |
| Median (IQR) | 0 (0) | 10 (16) | 10 (19) | 10 (17) | 10 (19) | 9.0 (20) | 9.0 (21) | 8.0 (24) | 9.5 (23) |  |

| **(b) Central subfoveal thickness in microns** | | | | | | | | | | |
| --- | --- | --- | --- | --- | --- | --- | --- | --- | --- | --- |
|  | DMO | | | | | | | | | |
|  | Baseline (N=2107) | Month 6 (N=1609) | Month 12 (N=1567) | Month 18 (N=1233) | Month 24 (N=1020) | Month 30 (N=962) | Month 36 (N=866) | Month 48 (N=715) | Month 60 (N=540) |  |
| **Central subfoveal thickness (CST; µm)** |  |  |  |  |  |  |  |  |  |  |
| Mean (SD) | 420 (120) | 320 (100) | 320 (99) | 330 (110) | 320 (100) | 320 (110) | 320 (110) | 330 (120) | 320 (120) |  |
| Median (IQR) | 400 (140) | 300 (130) | 290 (120) | 300 (140) | 300 (130) | 300 (130) | 300 (130) | 300 (130) | 290 (130) |  |
| **Change in CST from baseline** |  |  |  |  |  |  |  |  |  |  |
| Mean (SD) | 0 (0) | -100 (120) | -110 (130) | -100 (150) | -110 (150) | -100 (150) | -110 (160) | -100 (160) | -110 (150) |  |
| Median (IQR) | 0 (0) | -88 (130) | -93 (150) | -89 (160) | -95 (170) | -94 (170) | -93 (170) | -95 (190) | -100 (170) |  |
|  | CRVO | | | | | | | | | |
|  | Baseline (N=413) | Month 6 (N=359) | Month 12 (N=366) | Month 18 (N=298) | Month 24 (N=245) | Month 30 (N=216) | Month 36 (N=179) | Month 48 (N=126) | Month 60 (N=75) |  |
| **Central subfoveal thickness (CST; µm)** |  |  |  |  |  |  |  |  |  |  |
| Mean (SD) | 530 (160) | 300 (140) | 300 (140) | 290 (120) | 290 (130) | 290 (130) | 270 (110) | 270 (100) | 270 (110) |  |
| Median (IQR) | 530 (210) | 250 (110) | 250 (100) | 240 (100) | 240 (110) | 250 (97) | 240 (100) | 240 (89) | 240 (110) |  |
| **Change in CST from baseline** |  |  |  |  |  |  |  |  |  |  |
| Mean (SD) | 0 (0) | -210 (210) | -210 (220) | -230 (200) | -230 (200) | -240 (210) | -270 (200) | -270 (200) | -260 (170) |  |
| Median (IQR) | 0 (0) | -220 (270) | -210 (280) | -230 (280) | -240 (280) | -250 (290) | -260 (270) | -250 (240) | -260 (200) |  |
|  | BRVO | | | | | | | | | |
|  | Baseline (N=757) | Month 6 (N=620) | Month 12 (N=627) | Month 18 (N=500) | Month 24 (N=389) | Month 30 (N=362) | Month 36 (N=293) | Month 48 (N=197) | Month 60 (N=134) |  |
| **Central subfoveal thickness (CST; µm)** |  |  |  |  |  |  |  |  |  |  |
| Mean (SD) | 460 (140) | 290 (110) | 290 (100) | 290 (99) | 280 (110) | 280 (110) | 280 (100) | 290 (100) | 300 (100) |  |
| Median (IQR) | 440 (180) | 260 (100) | 260 (100) | 260 (100) | 250 (100) | 250 (95) | 260 (85) | 270 (110) | 270 (110) |  |
| **Change in CST from baseline** |  |  |  |  |  |  |  |  |  |  |
| Mean (SD) | 0 (0) | -150 (170) | -150 (170) | -150 (160) | -160 (180) | -160 (180) | -160 (180) | -150 (180) | -150 (170) |  |
| Median (IQR) | 0 (0) | -140 (220) | -140 (210) | -120 (220) | -140 (240) | -150 (240) | -150 (250) | -130 (240) | -150 (200) |  |

**Supplementary Table 1. Six-monthly change in visual acuity and central subfoveal thickness from baseline up to five years following anti-VEGF initiation.** Mean, median, standard deviation (SD), and interquartile range (IQR) are shown for (a) visual acuity in Early Treatment Diabetic Retinopathy Study letters (ETDRS) letters and (b) central subfoveal thickness (µm).
