# Supplementary material for: Visual and anatomical failure of anti-VEGF therapy for retinal vascular diseases: a survival analysis of real-world data
Source: Eye (Lond). 2024 Dec 10;39(5):977–85. doi: 10.1038/s41433-024-03529-9 (PMC11933433; doi:10.1038/s41433-024-03529-9)
Supplement: Supplementary file 2 — Supplementary Table 2. Baseline features stratified by anti-VEGF therapy treatment failure. [file 41433_2024_3529_MOESM2_ESM.docx]

|  | Treatment responsive (N=1598) | Treatment failure (N=1679) | Overall (N=3277) |
| --- | --- | --- | --- |
| **Gender** |  |  |  |
| Male | 896 (56.1%) | 979 (58.3%) | 1875 (57.3%) |
| Female | 701 (43.9%) | 699 (41.7%) | 1400 (42.7%) |
| **Age at recruitment** |  |  |  |
| Mean (SD) | 64 (13) | 65 (12) | 65 (12) |
| Median (IQR) | 64 (17) | 65 (15) | 65 (16) |
| **Ethnicity** |  |  |  |
| Afrocarribean | 145 (9.1%) | 179 (10.7%) | 324 (9.9%) |
| Caucasian | 424 (26.5%) | 470 (28.0%) | 894 (27.3%) |
| Chinese | 7 (0.4%) | 7 (0.4%) | 14 (0.4%) |
| Mixed | 16 (1.0%) | 19 (1.1%) | 35 (1.1%) |
| Southeast Asian | 420 (26.3%) | 462 (27.5%) | 882 (26.9%) |
| Unknown | 585 (36.6%) | 541 (32.2%) | 1126 (34.4%) |
| **Baseline Visual acuity (ETDRS letters)** |  |  |  |
| Mean (SD) | 54 (19) | 62 (15) | 58 (17) |
| Median (IQR) | 59 (24) | 65 (18) | 61 (20) |
| **Baseline Central foveal thickness (microns)** |  |  |  |
| Mean (SD) | 430 (150) | 440 (120) | 430 (140) |
| Median (IQR) | 400 (200) | 420 (150) | 410 (170) |
| **Intraretinal fluid (nl)** |  |  |  |
| Mean (SD) | 100 (110) | 99 (110) | 100 (110) |
| Median (IQR) | 63 (120) | 66 (110) | 64 (110) |
| **Subretinal fluid (nl)** |  |  |  |
| Mean (SD) | 230 (580) | 130 (420) | 180 (510) |
| Median (IQR) | 3.2 (130) | 0.58 (37) | 1.5 (67) |
| **Indication** |  |  |  |
| BRVO | 488 (30.6%) | 269 (16.0%) | 757 (23.1%) |
| CRVO | 258 (16.2%) | 155 (9.2%) | 413 (12.6%) |
| DMO | 851 (53.3%) | 1254 (74.7%) | 2105 (64.3%) |

**Supplementary Table 2. Baseline features stratified by anti-VEGF therapy treatment failure.**

Mean, median, standard deviation (SD), and interquartile range (IQR) are shown for demographic characteristics and clinical features at baseline. Treatment failure was taken to be one of either: visual acuity (VA) gain less than 5 early treatment diabetic retinopathy study (ETDRS) letters with central subfoveal thickness (CST) 325 µm or more at 2 consecutive visits; VA loss of 10 ETDRS letters and CST increase of 50 µm; or switch to steroid therapy) after starting anti-VEGF for DMO, CRVO, or BRVO. Baseline time point was taken to be time at initiation of intravitreal anti-VEGF therapy. Abbreviations: DME, diabetic macular edema; CRVO, central retinal vein occlusion; BRVO, branch retinal vein occlusion; VA, visual acuity; ETDRS, early treatment diabetic retinopathy study.
