# Supplementary material for: Visual and anatomical failure of anti-VEGF therapy for retinal vascular diseases: a survival analysis of real-world data
Source: Eye (Lond). 2024 Dec 10;39(5):977–85. doi: 10.1038/s41433-024-03529-9 (PMC11933433; doi:10.1038/s41433-024-03529-9)
Supplement: Supplementary file 3 — Supplementary Fig. 1. CONSORT flow diagram [file 41433_2024_3529_MOESM3_ESM.pdf]

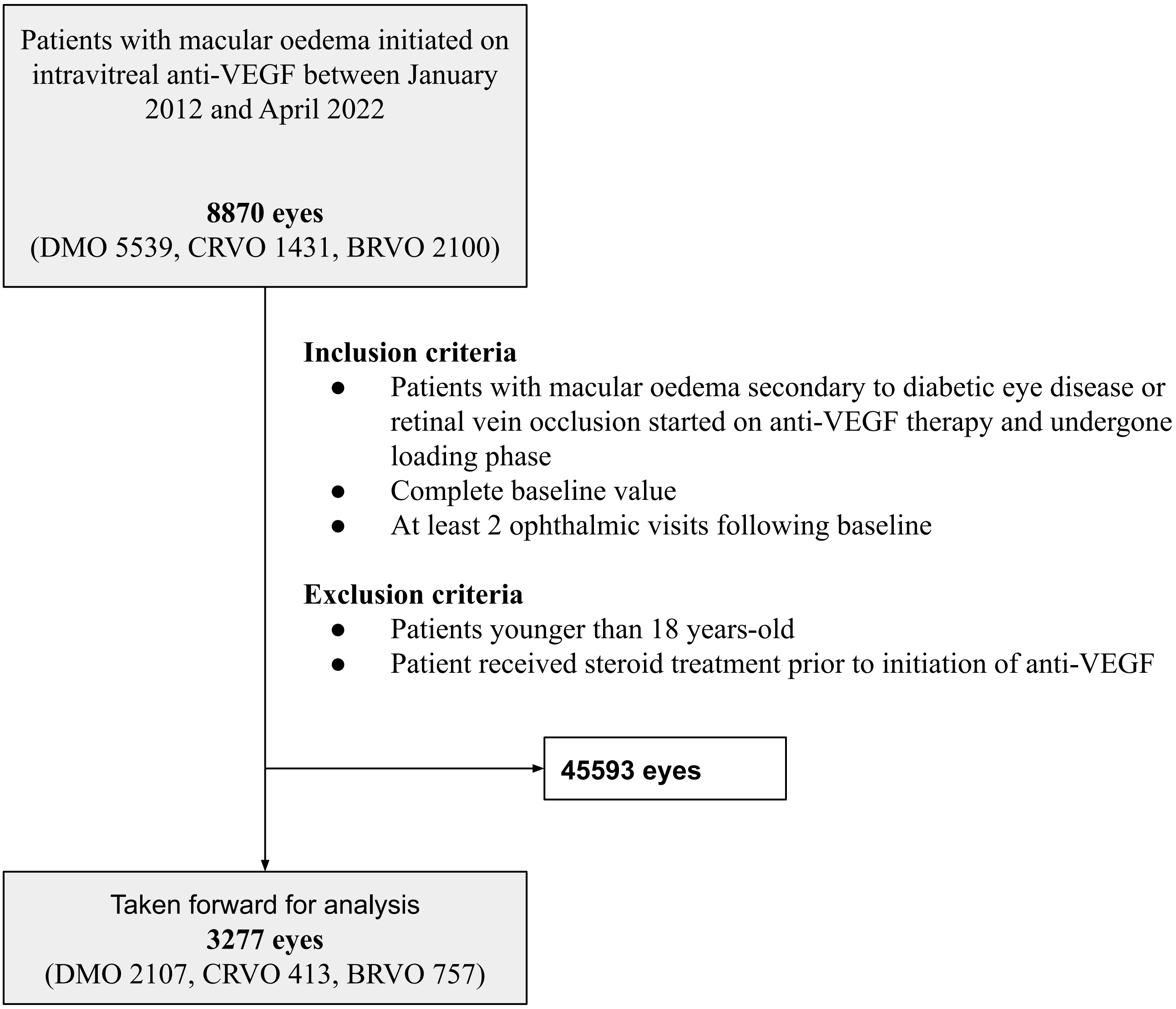
Patients with macular oedema initiated on intravitreal anti-VEGF between January 2012 and April 2022
8870 eyes
(DMO 5539, CRVO 1431, BRVO 2100)
Inclusion criteria
Patients with macular oedema secondary to diabetic eye disease or retinal vein occlusion started on anti-VEGF therapy and undergone loading phase
Complete baseline value
At least 2 ophthalmic visits following baseline
Exclusion criteria
Patients younger than 18 years-old
Patient received steroid treatment prior to initiation of anti-VEGF
45593 eyes
Taken forward for analysis
3277 eyes
(DMO 2107, CRVO 413, BRVO 757)
