# Supplementary figures and images for: Visual and anatomical failure of anti-VEGF therapy for retinal vascular diseases: a survival analysis of real-world data
Source: Eye (Lond). 2024 Dec 10;39(5):977–85. doi: 10.1038/s41433-024-03529-9 (PMC11933433; doi:10.1038/s41433-024-03529-9)

Heatmap of study eyes & VA measurements following anti-VEGF treatment. Up to 5 years

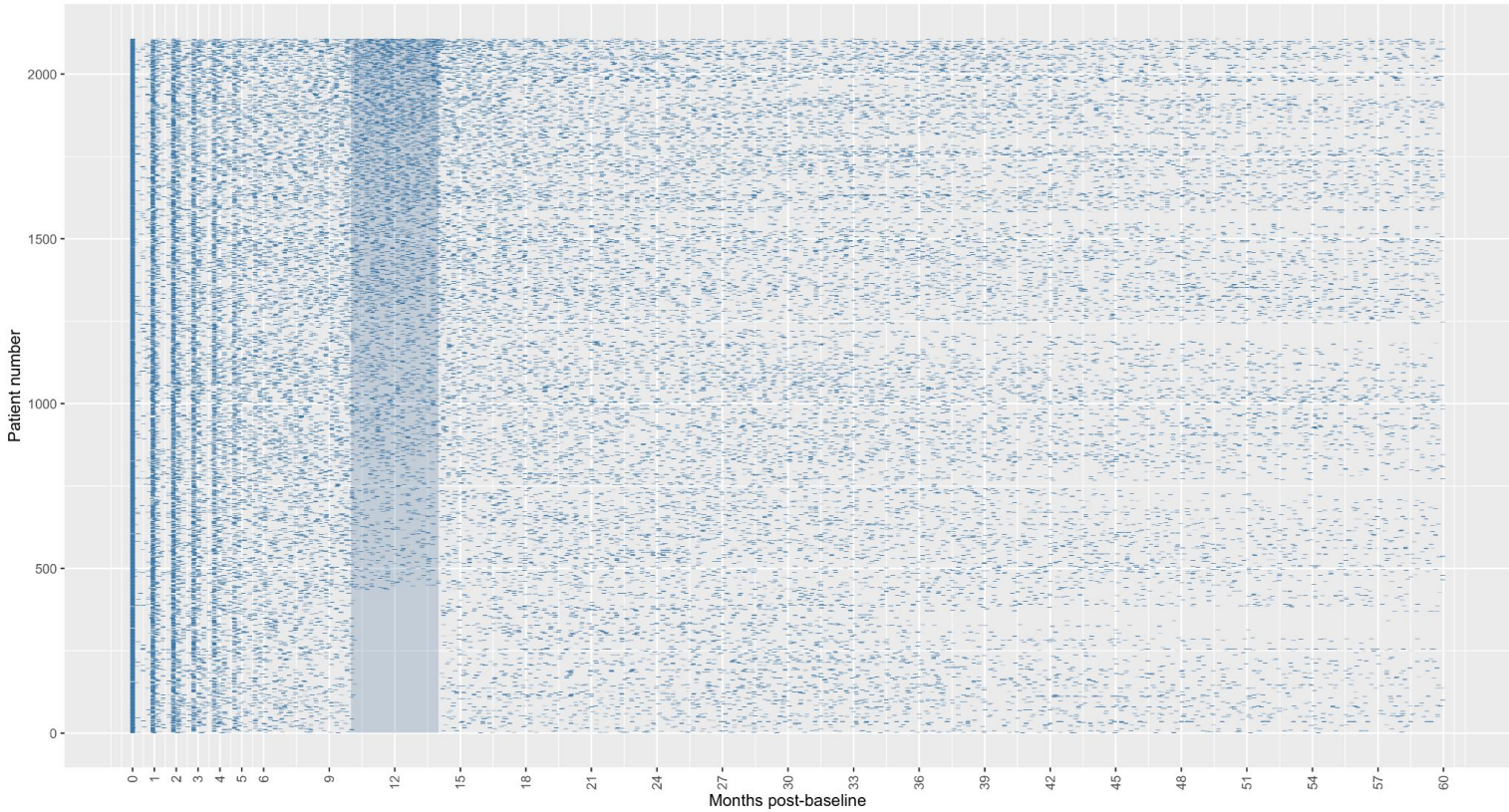

Supplement: Supplementary file 4 — Supplementary Fig. 2. Data capture at monthly timepoints [file 41433_2024_3529_MOESM4_ESM.pdf]
