# Supplementary material for: Visual and anatomical failure of anti-VEGF therapy for retinal vascular diseases: a survival analysis of real-world data
Source: Eye (Lond). 2024 Dec 10;39(5):977–85. doi: 10.1038/s41433-024-03529-9 (PMC11933433; doi:10.1038/s41433-024-03529-9)

(a) Visual acuity gain less than 5 ETDRS letters with CST 325 µm or more at 2 consecutive visits

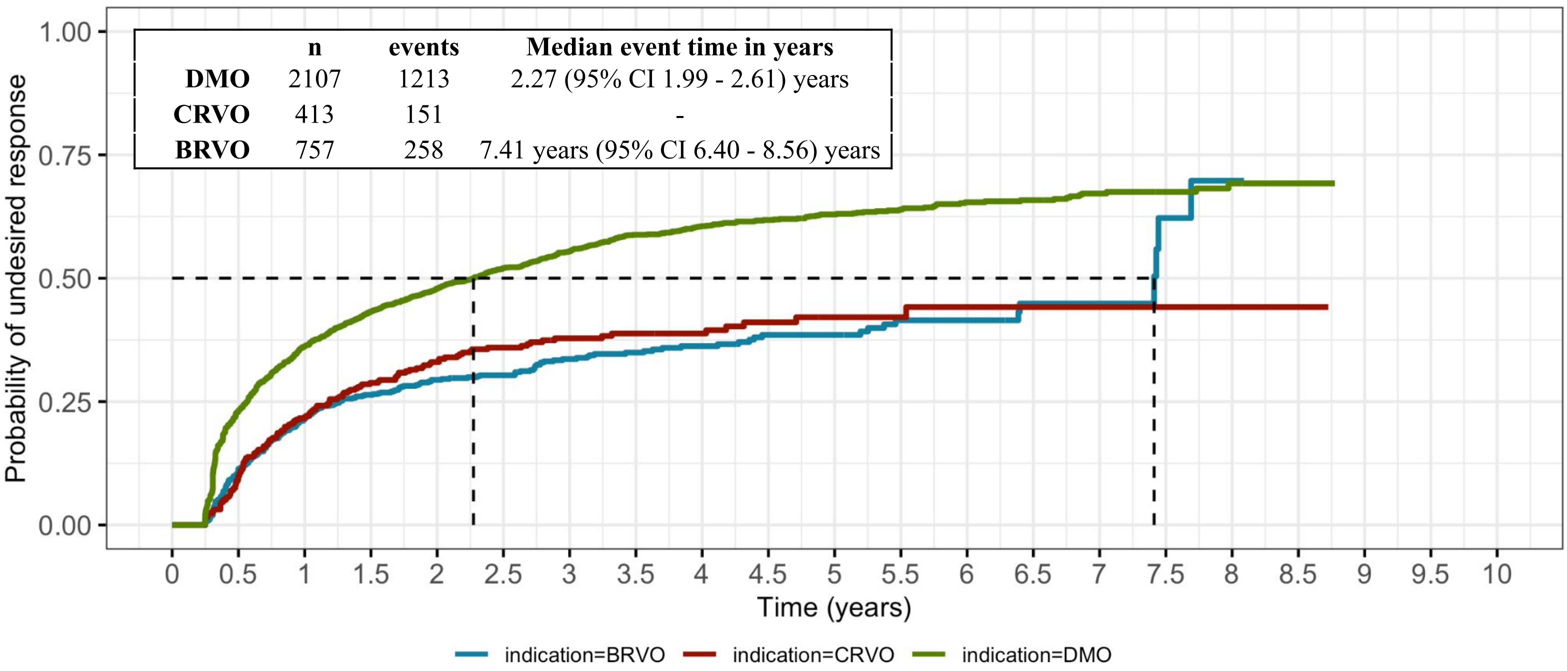

(b) CST increase by 50 µm and loss of at least 10 ETDRS letters from baseline

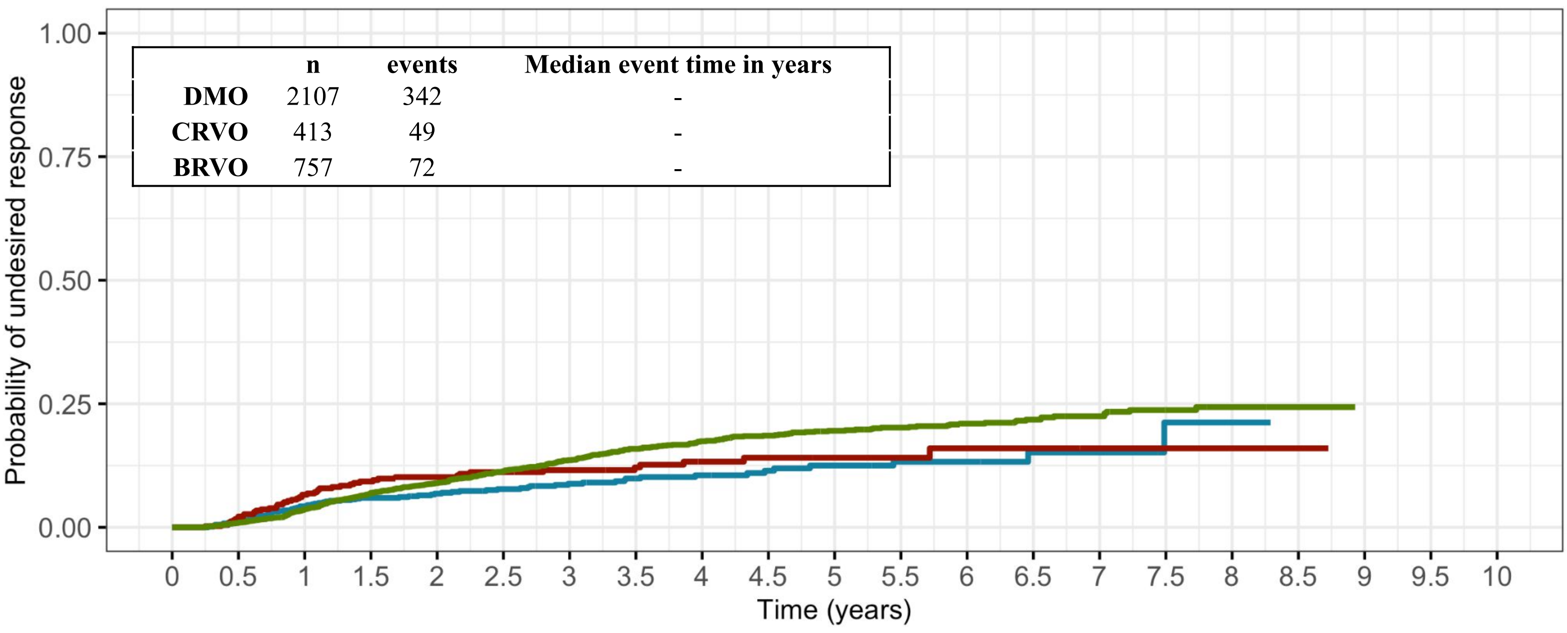

Supplement: Supplementary file 5 — Supplementary Fig. 3. Individual contributions of the treatment non-response criteria used in this model. [file 41433_2024_3529_MOESM5_ESM.pdf]
