# Supplementary material for: Visual and anatomical failure of anti-VEGF therapy for retinal vascular diseases: a survival analysis of real-world data
Source: Eye (Lond). 2024 Dec 10;39(5):977–85. doi: 10.1038/s41433-024-03529-9 (PMC11933433; doi:10.1038/s41433-024-03529-9)
Supplement: Supplementary file 6 — Supplementary Figs. and Tables Caption [file 41433_2024_3529_MOESM6_ESM.docx]

**Supplementary Figure 1. CONSORT flow diagram.** The cohort comprised patients with diabetic macular oedema (DMO) or macular oedema secondary to central retinal vein occlusion (CRVO) or branch retinal vein occlusion (BRVO) that were treatment-naïve and initiated on anti-VEGF therapy between January 2012 and April 2022. Exclusion criteria were: patients younger than 18 years-old; missing baseline metrics, including visual acuity, central subfoveal thickness, or retinopathy grading; incomplete loading (or induction) phase; and insufficient follow-up (fewer than two ophthalmic visits following initial injection with at least one falling beyond 6 months).

**Supplementary Figure 2. Data capture at monthly timepoints.**

Distinct clinical visits where visual acuity has been measured are plotted (blue dots). X-axis depicts time following initial intravitreal anti-VEGF and has been restricted to the first 5 years. Blue dots represent the 12 month timepoint with a 60 day margin. Distinct patients represented along the y-axis and arranged so that patients with a clinical visit within the 12 month timepoint are at top. Here it is demonstrated that an absent value does not suggest absence of follow-up. Of the 635 persons without a visual acuity (VA) measurement at the 12 month time point, 100% had a measurement following the 12 month period.

**Supplementary Figure 3. Individual contributions of the treatment outcome criteria used in this model.**

Kaplan-Meier modelling was carried out to independently estimate: (a) time to absence of vision improvement due to macular oedema (visual acuity [VA] gain less than 5 early treatment diabetic retinopathy study [ETDRS] letters with central subfoveal thickness (CST) 325 µm or more at 2 consecutive visits); and (b) visual decline due to macular oedema (VA loss of 10 ETDRS letters and CST increase of 50 µm). When individually considering the absence of VA improvement due to macular oedema (CST >325 µm and VA change from baseline <5 ETDRS letters at 2 consecutive visits), we recorded 1622 events with a median event time of 3.3 (95% CI 2.96 - 3.84) years. This was split across DMO (1213 events which corresponds to 58% patients of the DMO cohort), BRVO (258 events, 34 % of the BRVO cohort), and CRVO (151 events, 37% of the CRVO cohort). The median event time per cohort was 2.27 years for DMO, 7.41 years for BRVO, and >8.5 years post baseline for CRVO. Conversely, when focusing on the loss of VA attributable to increase in macular oedema (CST increase by 50 µm and loss of at least 10 ETDRS letters from baseline), we observed much fewer events, with only 463 (14%) of patients within the overall cohort meeting these criteria for vision loss. The median event time for this was longer than our observation period of 8.5 years. Analyses sub-stratified by each of the treatment indications (DMO, green; CRVO, red; BRVO, blue). Remaining numbers at risk shown in bottom panel.

**Supplementary Table 1. Six-monthly change in visual acuity and central subfoveal thickness from baseline up to five years following anti-VEGF initiation.** Mean, median, standard deviation (SD), and interquartile range (IQR) are shown for (a) visual acuity in Early Treatment Diabetic Retinopathy Study letters (ETDRS) letters and (b) central subfoveal thickness (µm).

**Supplementary Table 2. Baseline features stratified by anti-VEGF therapy treatment failure.**

Mean, median, standard deviation (SD), and interquartile range (IQR) are shown for demographic characteristics and clinical features at baseline. Treatment failure was taken to be one of either: visual acuity (VA) gain less than 5 early treatment diabetic retinopathy study (ETDRS) letters with central subfoveal thickness (CST) 325 µm or more at 2 consecutive visits; VA loss of 10 ETDRS letters and CST increase of 50 µm; or escalation to steroid therapy) after starting anti-VEGF for DMO, CRVO, or BRVO. Baseline time point was taken to be time at initiation of intravitreal anti-VEGF therapy. Abbreviations: DMO, diabetic macular oedema; CRVO, central retinal vein occlusion; BRVO, branch retinal vein occlusion; VA, visual acuity; ETDRS, early treatment diabetic retinopathy study.
